# Nearly Complete Genome Sequences of 12 Types of Human Rhinoviruses Isolated from Pediatric Inpatients in Fukushima, Japan

**DOI:** 10.1128/mra.00529-22

**Published:** 2022-07-13

**Authors:** Kazutaka Egawa, Masatoshi Kakizaki, Yohei Kume, Reiko Suwa, Miyuki Kawase, Takashi Ono, Mina Chishiki, Hisao Okabe, Sakurako Norito, Masatoki Sato, Hiroko Sakuma, Shigeo Suzuki, Mitsuaki Hosoya, Makoto Takeda, Koichi Hashimoto, Kazuya Shirato

**Affiliations:** a Department of Virology III, National Institute of Infectious Disease, Musashimurayama, Tokyo, Japan; b Department of Pediatrics, School of Medicine, Fukushima Medical University, Fukushima, Fukushima, Japan; c Hoshi General Hospital, Koriyama, Fukushima, Japan; d Ohara General Hospital, Fukushima, Fukushima, Japan; Portland State University

## Abstract

We reported nearly complete genomic sequences of 12 serotypes of human rhinoviruses (HRVs) isolated from pediatric inpatients in Fukushima, Japan using an air-liquid interface culture of human bronchial tracheal epithelial cells. We found that various serotypes of HRV circulated locally and simultaneously from 2018 to 2021.

## ANNOUNCEMENT

Human rhinoviruses (HRVs) belong to the family *Picornaviridae* and have a genome that consists of approximately 7.2 kb of positive-sense single-stranded RNA. There are three main species of HRVs (A, B, C) and more than 160 (sero)types (1, 2). Once the HRV types were distinguished by serological assays. Currently, they are distinguished by genetic methods due to the difficulty of virus propagation in cell cultures (3). The HRV genome comprises a 5′ untranslated region that contains an internal ribosome entry site, a single open reading frame, and a 3′ untranslated region (4). HRVs are a major cause of pediatric pneumonia following respiratory syncytial virus infection and sometimes cause severe acute respiratory infections in children (5–8). HRVs have also been detected in coinfections with acute respiratory pathogens (7–9), and coinfection with coronaviruses has recently been reported to affect the outcome of diseases by exacerbating or reducing virus infections (10, 11).

In this study, 12 HRVs were successfully isolated from specimens obtained from pediatric patients with severe acute respiratory infections in Fukushima, Japan, and their nearly complete genomic sequences were obtained (Table 1). Nasopharyngeal swabs were collected from 2018 to 2021, and those that were HRV positive by multiplex real-time PCR for respiratory viruses (12–14) were used for virus isolation. The air-liquid interface culture of human bronchial/tracheal epithelial cells (HBTEC-ALI) was prepared as described previously (15–17). At 7 or 11 days after inoculation onto HBTEC-ALI culture with specimens, cells were washed with a culture medium, and the presence of virus in cell wash was confirmed by real-time RT-PCR. The cell wash showed that HRV positive was stored as virus stock. Nucleic acids were extracted from virus stock using a QIAamp Viral RNA Mini kit (Qiagen, Hilden, Germany). Libraries for next-generation sequencing were prepared using a NEBNext Ultra II RNA Library Prep Kit for Illumina (New England Biolabs, Ipswich, MA, USA) following the manufacturer’s instructions. The indexed libraries were analyzed for 2 × 150 cycles on a DNBSEQ-G400 sequencer at Genewiz (South Plainfield, NJ, USA). Reads were trimmed and then *de novo* assembled using the CLC Genomics Workbench (v21.0.4, Qiagen) with default settings. The assembled sequences were analyzed on BLASTn (https://blast.ncbi.nlm.nih.gov/Blast.cgi?PROGRAM=blastn&BLAST_SPEC=GeoBlast&PAGE_TYPE=BlastSearch) and the sequence that showed the highest similarity was considered the HRV type of the assembled sequence. The coverage was calculated by mapping of reads to the assembled sequence.

**TABLE 1:** Details of the 12 human retroviruses (HRVs) isolated in this study

Name	Accession no.	Run data accession no.	Total reads	Total mapped read	Average of coverage	Length	GC%	Species and type	No. of registered complete genomic sequences^*a*^	Coinfection^*b*^	Cp value of specimen
Fukushima_H260_2018	LC699414	DRR374977	20,955,278	570,481	10,849.94	7,180	38.05	A78	40	PIV3, HBoV	38.13
Fukushima_H287_2018	LC699415	DRR374978	12,042,228	348,159	6,925.07	7,185	37.63	A24	20	RSVB, PIV3, HBoV	37.98
Fukushima_H504_2019	LC699416	DRR374979	19,252,620	542,834	10,822.21	7,122	38.92	A54	4	ADV2, hMPV	33.87
Fukushima_H555_2019	LC699417	DRR374980	23,129,996	608,521	12,230.00	7,150	38.83	A81	3	RSVB, ADV2, ADV4, hMPV	32.8
Fukushima_H561_2019	LC699418	DRR374981	12,473,136	830,017	15,718.68	7,150	38.39	A16	2	RSVB, PIV3, ADV4, HBoV	31.39
Fukushima_H581_2019	LC699419	DRR374982	17,532,206	1,938,266	37,427.98	7,189	39.07	A88	1	RSVA, PIV3, ADV2, HBoV	34.66
Fukushima_H681_2019	LC699420	DRR374983	17,366,078	526,938	10,336.42	7,143	38.83	A58	7		25.58
Fukushima_HR13_2020	LC699421	DRR374984	9,147,658	474,174	9,396.38	7,146	37.59	A21	4		24.21
Fukushima_OR4-2_2020	LC699422	DRR374985	18,567,126	1,032,272	20,467.83	7,142	38.46	A60	4		24.86
Fukushima_OR65_2020	LC699423	DRR374986	13,280,800	99,660	1,994.51	7,094	39.02	A80	10	HBoV	29.57
Fukushima_OR274_2021	LC699424	DRR374987	20,955,278	570,481	10,849.94	7,067	38.05	A1	207	RSVA, ADV2, HBoV	34.95
Fukushima_H399_2018	LC699425	DRR374988	15,474,968	6,254	127.50	7,041	43.15	C3	36	RSVB	27.78

a Number of nearly complete HRV genomic sequences in GenBank other than those obtained in this study.

b Coinfection was determined by multiplex real-time PCR assays for respiratory viruses. PIV, parainfluenzavirus; HBoV, human bocavirus; RSV, respiratory syncytial virus; ADV, adenovirus; hMPV, human metapneumovirus.

Among the 12 HRV isolates, three were mono-infections and nine were coinfections with other viruses (Table 1). Using real-time RT-PCR tests to detect HRVs, the Cp values ranged from 24.21 to 38.13. The Cp values in the coinfection samples were high. The type of each isolate was different. Among them, the number of registered complete sequences was very small for several types (Table 1). The 12 isolates and their genomic sequence information will be helpful to study virus characteristics, such as serological responses, in rare types.

### Data availability.

The 12 nearly complete HRV genome sequences have been deposited in GenBank under accession numbers LC699414, LC699415, LC699416, LC699417, LC699418, LC699419, LC699420, LC699421, LC699422, LC699423, LC699424, and LC699425 (Table 1). The raw reads have been deposited under BioProject number PRJDB13572. Each run data set has been deposited in DDBJ under accession number DRR374977, DRR374978, DRR374979, DRR374980, DRR374981, DRR374982, DRR374983, DRR374984, DRR374985, DRR374986, DRR374987, and DRR374988.
